# The crystal structure of the EspB-EspK virulence factor-chaperone complex suggests an additional type VII secretion mechanism in *Mycobacterium tuberculosis*

**DOI:** 10.1016/j.jbc.2022.102761

**Published:** 2022-12-01

**Authors:** Abril Gijsbers, Mathias Eymery, Ye Gao, Isabella Menart, Vanesa Vinciauskaite, Dritan Siliqi, Peter J. Peters, Andrew McCarthy, Raimond B.G. Ravelli

**Affiliations:** 1 Division of Nanoscopy, Maastricht Multimodal Molecular Imaging Institute (M4i), Maastricht University, Maastricht, the Netherlands; 2 European Molecular Biology Laboratory, Grenoble, France; 3 Istituto di Cristallografia, Consiglio Nazionale delle Ricerche, Bari, Italy

**Keywords:** chaperone, EspB, EspK, ESX-1, mycobacteria, PDB, Protein Data Bank, SAXS, small-angle X-ray scattering, SEC, size-exclusion chromatography, TB, tuberculosis

## Abstract

Pathogenic species from the *Mycobacterium* genus are responsible for a number of adverse health conditions in humans and animals that threaten health security and the economy worldwide. Mycobacteria have up to five specialized secretion systems (ESX-1 to ESX-5) that transport virulence factors across their complex cell envelope to facilitate manipulation of their environment. In pathogenic species, these virulence factors influence the immune system’s response and are responsible for membrane disruption and contributing to cell death. While structural details of these secretion systems have been recently described, gaps still remain in the structural understanding of the secretion mechanisms of most substrates. Here, we describe the crystal structure of *Mycobacterium tuberculosis* ESX-1 secretion-associated substrate EspB bound to its chaperone EspK. We found that EspB interacts with the C-terminal domain of EspK through its helical tip. Furthermore, cryogenic electron microscopy, size exclusion chromatography analysis, and small-angle X-ray scattering experiments show that EspK keeps EspB in its secretion-competent monomeric form and prevents its oligomerization. The structure presented in this study suggests an additional secretion mechanism in ESX-1, analogous to the chaperoning of proline-glutamate (PE)–proline-proline-glutamate (PPE) proteins by EspG, where EspK facilitates the secretion of EspB in *Mycobacterium* species.

Human tuberculosis (TB) is a widespread infectious disease, caused by the bacillus *Mycobacterium tuberculosis*. In 2019, 10 million cases and 1.4 million deaths have been reported due to TB, making it the leading cause of death from a single infectious agent and a colossal burden on global human health (1). The COVID-19 pandemic has had a major impact on the ongoing TB crisis. For the first time in more than a decade, TB mortality has increased (2). It is projected that the COVID-19 and TB syndemic have resulted in a 5 year setback in terms of mortality from TB and a 9 year setback in terms of TB detection (3). COVID-19 has disrupted TB services worldwide (4, 5) and caused new hazardous risks associated with TB/COVID-19 coinfection (6). Whereas, new vaccines have been developed for COVID-19 within a year, TB vaccination still relies on the Bacille Calmette-Guérin (BCG) vaccine, which is over a century old. The global TB crisis calls for development of new drugs and vaccines that efficiently target *M. tuberculosis*, which requires identification and characterization of treatment targets in the *M. tuberculosis* infection cycle.

Several important aspects of *M. tuberculosis* infection have been elucidated. A unique aspect of *M. tuberculosis* infection is the ability to escape lytic degradation in the phagolysosomes of human alveolar macrophages (7, 8), which phagocytose tubercle bacilli upon infection (9). The lack of this ability in nonpathogenic mycobacteria leads to lytic degradation (10), indicating that this event is crucial for intracellular survival and persistent infection by pathogenic mycobacteria, including *M. tuberculosis* (7). The translocation of tubercle bacilli occurs through the permeabilization of the host phagosome and has been directly linked to the ESAT-6 secretion systems (ESX), specifically ESX-1 (10, 11).

ESX secretion systems are prominent effectors of mycobacterial virulence and participate in substrate secretion across the mycobacterial envelope (11). Five paralogous ESX systems (ESX-1 to -5), unable to complement each other’s function, have been identified across mycobacteria (12). While ESX-1 has been implicated in substrate transport across the inner membrane (12) and has been directly linked to phagosome permeabilization by a contact-dependent mechanism (10, 13), the details of its structure and function remain elusive. Since the five ESX systems show high similarity in genetic organization and sequence conservation (14), it could be expected that they are unified by a similar architecture. Hence, the recent high-resolution structures of ESX-3 (15, 16) and ESX-5 (17, 18) might provide clues into the architecture and function of ESX-1.

The core secretion machinery, formed by ESX-conserved components (Ecc), EccB, EccC, EccD, and EccE (12, 19), and stabilized by the subtilisin-like serine protease mycosin MycP (20, 21), secretes several classes of substrates (22). These include proteins of the WxG100 superfamily composed of 100 amino acids and a highly conserved WxG motif (23); proline-glutamate (PE) and proline-proline-glutamate (PPE) proteins secreted as heterodimers (24), and alanine-rich proteins (25), the transport of which is facilitated by a conserved YxxxD/E motif (26). It is currently known that ESX-1 is located in the inner membrane. One could expect ESX-1 to extend to the outer membrane; however, this remains to be shown (22, 27). Several ESX-1 substrates have been suggested to form the missing components of the extended ESX-1 machinery (28, 29), but additional studies are necessary to provide a more complete knowledge of ESX-1 secretion.

One of these substrates, the ESX-1 secretion-associated protein B (EspB; Rv3881c), has been postulated as a structural component of the extended ESX-1 machinery (29, 30). Deletion of *espB* attenuates *Mycobacterium marinum* survival during infection and its cytotoxicity activity (31, 32, 33), which emphasizes that EspB is an essential component of ESX-1. While EspB belongs to the PE/PPE heterodimer family (34, 35), it differs from other members of the family in that EspB is a single polypeptide that contains the bipartite secretion signal sequence, formed by WxG and YxxxD/E motifs (34). The latter, contained within the “export arm,” is hypothesized to be involved in the interaction to EccCb_1_, targeting EspB for secretion (34). Once secreted across the inner mycobacterial membrane, EspB oligomerizes into a cylinder-like heptamer (30, 34, 35), favored by the removal of its C-terminal region and the acidic pH environment of the phagosomal lumen (29). Oligomerization of EspB is driven by the PE/PPE N-terminal region (34) and mediated by the ESX-1 core complex MycP_1_ protease, which cleaves the full-length 48-kDa precursor EspB monomer at its C-terminal domain (36), leaving a mature 38-kDa isoform to be secreted (32). The resulting EspB heptamer has been proposed to form channel-like structures, enabling other ESX-1 secreted substrates to pass through and beyond the inner mycobacterial envelope (29). Improving our understanding of EspB secretion and function, as well as the regulatory mechanism behind its secretion could reveal how EspB completes the ESX-1 core secretion machinery and participates in substrate secretion.

To date, it is recognized that the secretion signals are not enough to target the substrate to its specific secretion machinery (26) and that chaperones are needed to perform this role (37, 38). It is expected that EspB secretion, like other substrates, also relies on a chaperone to deliver it to the correct secretion machinery. As EspB secretion does not depend on EspG (32), unlike its structurally homologs PE/PPE pairs, it is likely that EspB utilizes a different secretion pathway. EspB interacts with EspK (Rv3879c) (32), which is encoded by a gene in the RD1 locus downstream of EspB (11). Lim *et al*. (39) write that a direct physical interaction between *M. tuberculosis* EspB and EspK requires the WxG motif of EspK, which is located in its N-terminus. Others suggested that only the EspK C-terminal domain interacts with the EspB N-terminus in the cytosol, which is then delivered to EccCb_1_ for secretion (32). Structural analysis of EspK at low resolution combined with structure predictions identified the presence of N- and C-terminal domains with independent behavior, connected by an unstructured low-complexity linker (40). However, the detailed structure of EspK and its role have yet to be determined, especially its interaction with EspB, and possibly other ESX-1 proteins.

Here, we present small-angle X-ray scattering (SAXS) and crystal structures of the EspK C-terminal domain bound to the N-terminal domain of EspB, both from *M. tuberculosis*. EspK binds to the EspB helical tip, analogous to PE–PPE–EspG interactions. EspK also binds to the PE-PPE loop of EspB: this loop is known to undergo conformational changes to allow for postsecretion oligomerization of EspB. EspK binding to EspB prevents EspB oligomerization not only by steric hindrance but also by locking the PE-PPE loop, preventing it to adapt the more open conformation that is required for EspB oligomerization. Cryo-EM, size-exclusion chromatography (SEC), and SAXS experiments show fewer EspB oligomers when EspK was present, suggesting that EspK binds EspB in a secretion-competent monomeric state and act as a chaperone by preventing premature oligomerization. Analogous to what we see in the PE–PPE–EspG secretion mechanism and based on our results, we propose that EspB is secreted through a new secretion route for the ESX-1 system that is mediated by EspK, where the chaperone keeps EspB in a secretion-competent state and directs the protein to the machinery.

## Results

### EspB and EspK form a heterodimer in solution

The interaction between EspB and EspK was originally reported using a two-hybrid system and confirmed by pull-down assays (32). We first characterized this interaction for our *M. tuberculosis* constructs (Table S1). SEC profiles of the proteins alone and incubated together confirm that the C-terminal domain of EspK interacts with the N-terminal region of EspB, shown by the presence of a peak eluting earlier than the protein alone (residues 7–278, Fig. S1). Testing three different EspB constructs with longer C-terminal regions (EspB_7-278_, EspB_2-348_, and EspB_2-460_; Table S1) showed complex formation. To gain insight how the complex is formed, we first performed SAXS on the EspB constructs (Table S2). As expected, the EspB constructs showed differences in parameters like maximum dimension (*Dmax*) (Fig. S2*A*), radius of gyration (*R*_*g*_), and the Porod exponent (level of compactness); higher flexibility was observed with longer C-terminal regions, characterized by a change in the shape of the dimensionless Kratky plot, from a Gaussian distribution (EspB_7-278_) to a plateau (EspB_2-460_) (Fig. S2*B*). SAXS experiments in the presence of EspK_484-729_ provided similar *R*_*g*_ values for the full-length monomeric EspB_2-460_, independent on whether EspK was present or not (Table S2). This suggests that EspK_484-729_ does not interact with the flexible C-terminal region. For the shortest monomeric EspB fragment (EspB_7-278_), *R*_*g*_ as well as the estimated molecular mass are significantly larger in the presence of EspK (Table S2) and indicate the formation of a complex with a 1:1 stoichiometry. The experimental SAXS signal obtained from an EspB_2-348_–EspK_484-729_ mixture compares well (Fig. S2*D*) to the calculated SAXS curve based on models made from the crystal structure described later.

### EspB interacts with the C-terminal domain of EspK through the helical tip

**Figure 1:**
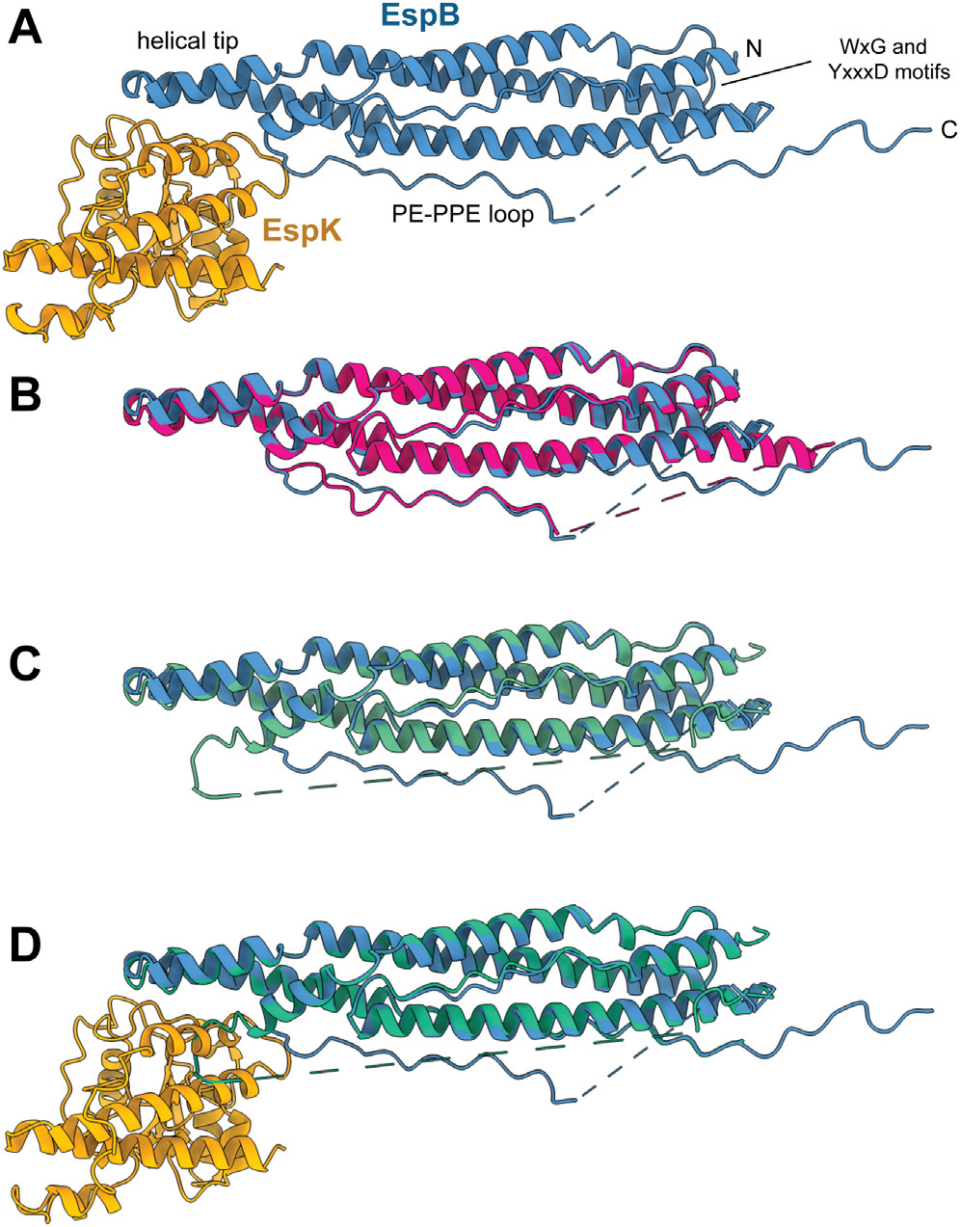
**EspB interacts with EspK through the helical tip and PE-PPE loop.***A*, EspB_2-300_ (*blue*) and EspK_484-729_ (*gold*) from *M. tuberculosis* complex model. Structural comparison between EspB from this study and (*B*) crystal structure of monomeric EspB (*magenta*, PDB ID 4XXX) or (*C*) from oligomer structure (*green*, PDB ID 7P13). *D*, comparison between EspB in an oligomeric state (*green*) and EspB–EspK complex (*blue*-*gold*), highlighting the steric hindrance of the end of the PE-PPE loop. PDB, Protein Data Bank.

To understand how these two proteins interact precisely, we solved the crystal structure of the complex. An initial low resolution structure was obtained with EspB_2-348_ and EspK_484-729_ from *M. tuberculosis*, on the basis of which a new shorter EspB construct was designed (residues 2–300) while retaining important contact points in the crystal packing. This improved the crystal diffraction and led to a final anisotropic resolution of 2.3 Å (Table 1). Figure 1*A* shows the overall structure of the EspB_2-300_–EspK_484-729_ complex: EspB is shown in blue and EspK in gold. The crystal structure covers residues V9 to P297 for EspB, missing E87–S112 from the PE-PPE loop, whereas all residues could be built for EspK_484-729_. The latter binds to the helical tip of EspB, as well as to the end of the PE-PPE loop of EspB (see more later).

**Table 1:** Crystallographic data collection and refinement statistics of *M. tuberculosis* EspB–EspK

Crystal	Native	S-SAD	Hg-derivative
Wavelength	0.976	2.066	1.008
Space group	P6_1_22	P6_1_22	P6_1_22
Cell constants (Å) a, b, c	101.6, 101.6, 377.0	99.5, 99.5, 375.8	99.9, 99.9, 376.93
α, β, γ (°)	90, 90, 120	90, 90, 120	90, 90, 120
Resolution range (Å)	85.7–2.3 (2.6–2.3)^a^	86.2–2.9 (3.3–2.9)^a^	86.5–2.8 (3.2–2.8)^a^
Ellipsoidal^b^ (direction)	3.1 (0.894 a∗- 0.447 b∗)	3.6 (0.894 a∗- 0.447 b∗)	4.0 (0.894 a∗- 0.447 b∗)
	3.1 (b∗)	3.6 (b∗)	4.0 (b∗)
	2.3 (c∗)	2.9 (c∗)	2.8 (c∗)
Unique reflections	28,129 (1406)	16,171 (810)	13,712 (687)
Multiplicity	6.2 (8.5)	19.5 (18.2)∗	5.8 (6.0)∗

Completeness (%)			

Spherical	53.2 (9.0)	65.5 (11.7)	47.7 (7.1)
Ellipsoidal^c^	94.9 (88.4)	94.9 (74.4)	93.7 (77.9)
R_pim_ (%)	3.4 (38.0)	10.5 (69.9)	9.6 (62.1)
Mean < I/σ(I) >	11.7 (1.9)	10.0 (2.0)	9.4 (1.9)

Refinement program	Refmac 5.8.0267		

R, R_*free*_	0.235, 0.279		
R_*free*_ test set	1444 reflections (5.13%)		
Wilson B-factor (Å^2^)	63.5		
Anisotropy	0.103		
F_*o*_, F_*c*_ correlation	0.92		
Total number of atoms	3930		
<B> all atoms (Å^2^)	84.0		

a Values in parentheses are for the highest resolution shell.

b Data truncated by STARANISO to remove poorly measured reflections affected by anisotropy. The resolution limits for three directions in reciprocal space in STARANISO are indicated here.

c The anisotropic completeness was obtained by least squares fitting an ellipsoid to the reciprocal lattice points at the cutoff surface defined by a local mean I/σ(I) threshold of 1.2.

The structure of EspB in complex with EspK is similar to that of EspB alone (35) (Protein Data Bank [PDB] ID: 4XXX rmsd 1.0 Å for 239 Cα atoms); it contains alpha helical elements arranged in a paperclip-like structure, with one end the helical tip and on the other end the WxG and YxxD motifs (Fig. 1*A*). Figure 1*B* shows an overlay between EspB_2-300_ (blue) of our complex structure compared to the crystal structure of EspB_7-278_ (magenta) (PDB ID: 4XXX). The largest Cα positional differences are found within the helical tip (residues 227–246), the start of the PE-PPE loop (residues 82–86) and the end of the PE-PPE loop (residues 125–130). Our crystal structure shows residues 279 to 297, which were absent in the construct used to determine the crystal structure of EspB_7-278_. Figure 1*C* shows an overlay between EspB_2-300_ (blue) of our complex structure and a monomer from the heptameric cryo-EM structure of EspB_2-287_ (green) (29) (PDB ID: 7P13, rmsd 1.99 Å for 244 Cα atoms). Also here, the largest differences in Cα positions are found in the helical tip (residues 227–246) and the end of the PE-PPE loop (residues 122–130), whereas the start of the PE-PPE loop is more similar (residues 82–86). The position of the end of the PE-PPE loop as observed within the heptameric EspB structure (green), would be incompatible with the binding of EspK_484-729_ (gold), as these overlap (Fig. 1*D*).

### The C-terminal domain of EspK has a sea snail–like structure

**Figure 2:**
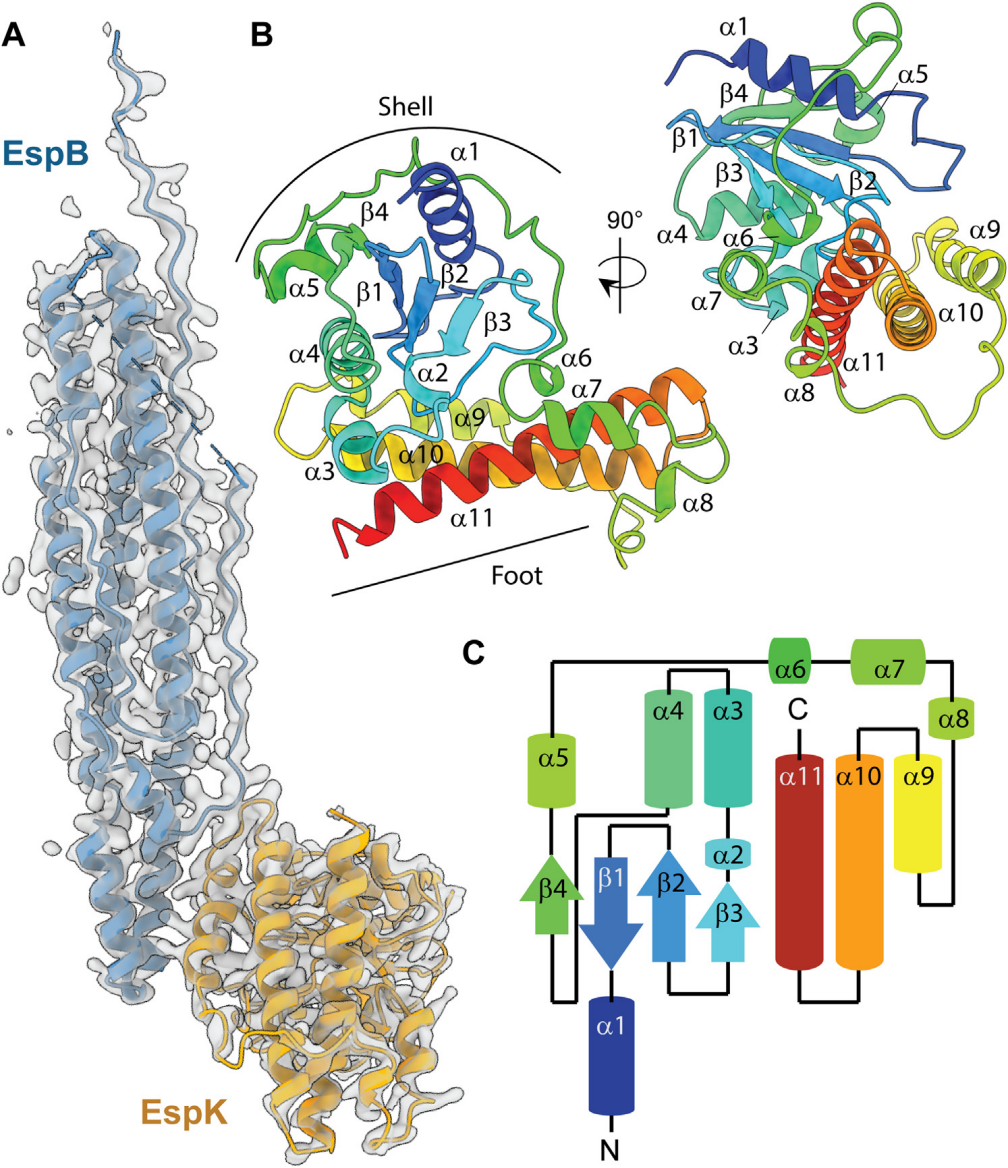
**EspK C-terminal domain overall structure**. *A*, map and model representation of EspB_2-300_ and EspK_484-729_ complex. *B*, ribbon representation of EspK structure, where secondary elements are labeled α1–α11 and β1–β4. *C*, topology diagram displays overall distribution of secondary structure.

Hitherto, no experimental PDB structure had been known for EspK. A combination of molecular replacement and SAD phasing allowed us to refine a model for EspB and to build and refine a new model for EspK_484-729_ (Fig. 2*A*). Our experimental structure of EspK_484-729_ (determined prior to the release of the AlphaFold database (41)) corresponds remarkably well with the C-terminal part of the AlphaFold model (AF-P9WJC1-F1, rmsd 0.97 Å for 246 Cα atoms). Figure 2*B* gives a ribbon representation, displaying 11 α-helices and four β-strands arranged in the order α1-β1-β2- β3-α2-α3-α4-β4-α5-α6-α7-α8-α9-α10-α11. The structure resembles a sea snail, consisting of two domains. The shell consists of an antiparallel β-sheet with five peripheral α helices. The foot of the snail is formed by a compact helix bundle of three α helices with a loop containing three short helices connecting the foot with the shell (Fig. 2*C*).

A Dali search of the built model of EspK_484-729_ revealed one best hit (Z-score 10.3, rmsd 3.7 Å, 160 aligned amino acids): PDB ID 5IMU. This is a fragment (residues 184–410) of the conserved hypothetical protein Rv3899c from *M. tuberculosis*. We had identified this protein previously to be potentially related to EspK (40). Its gene is located next to the ESX-2 locus, a paralog of the ESX-1 system EspK belongs to. The fragment of Rv3899c also consists of an α/β/α sandwich domain flanked by a helix bundle, but is overall a bit more compact. A Dali search against the AlphaFold database of *M. tuberculosis* reveals two more genes with a fragment that resembles the 3D fold EspK_484-729_: Rv0029 and Rv2082. Both are conserved hypothetical proteins with a domain that is structurally nearest to the fragment of Rv3899c. It is noteworthy that all our Dali searches with EspK_484-729_ only revealed mycobacterial proteins, suggesting this to be a specific fold for that genus.

### The interaction between EspB and EspK

**Figure 3:**
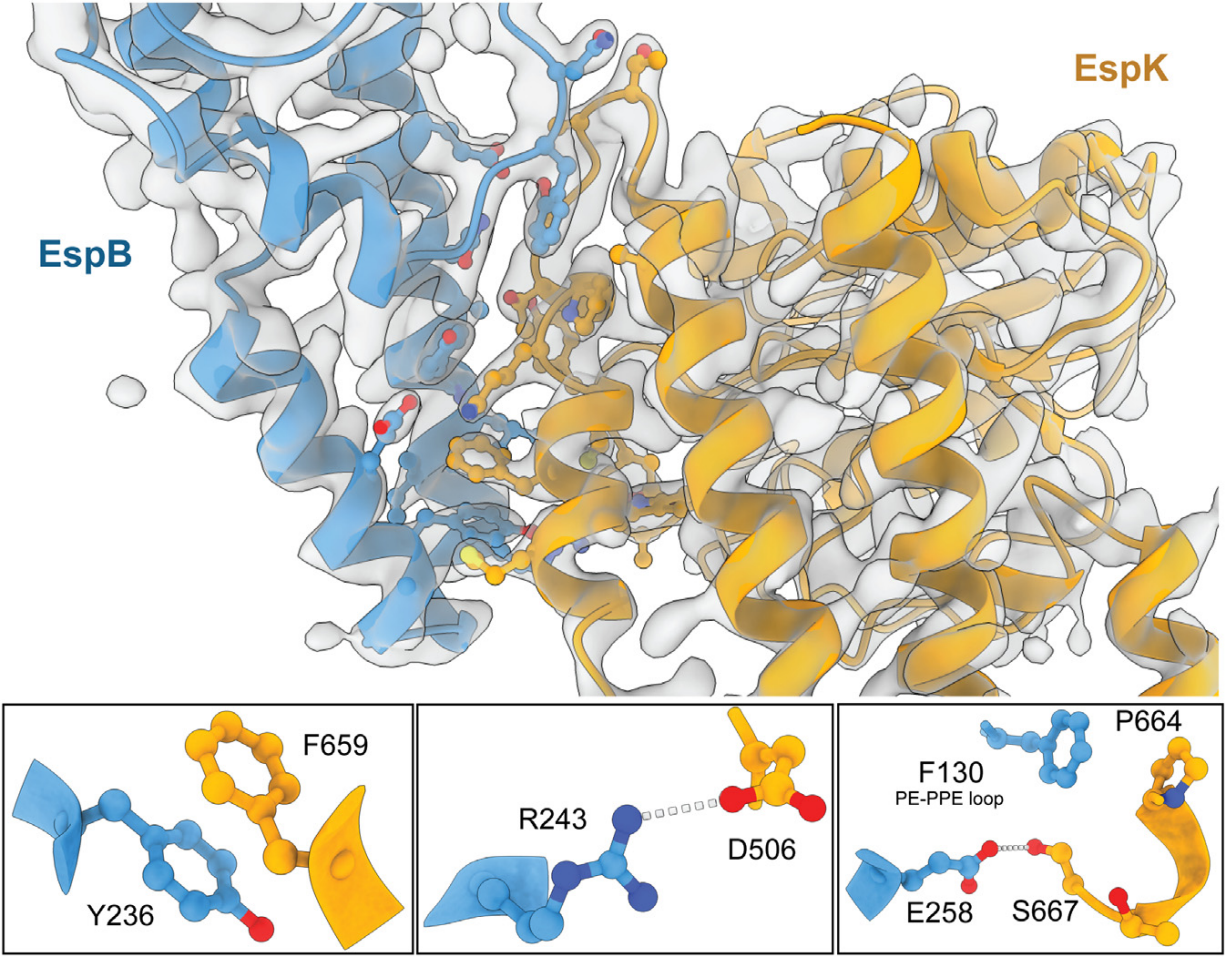
**Interaction between EspB and EspK.** Map and model representation of EspB_2-300_ and EspK_484-729_ complex with residues involved in the interaction depicted by stick and balls. Three examples of such interactions are displayed below. The distances for the polar interaction between OD1 of D506 and NH2 of R243 is 3.0 Å, whereas this distance is 2.6 Å for the interaction between OG of S667 and OE2 of E258.

EspK binds to the helical tip of EspB (Figs. 1*A* and 2*A*), similar to what has been described for PE-PPEs and their chaperone EspG (37, 42, 43, 44). The interaction occurs between α6–7 and the PE-PPE loop of EspB and α9–10 of EspK. Analysis of the EspB-EspK interface by PISA (45) gave an interface area of 652 Å^2^, ΔG of -11.7 kcal/mol, and a relative low complexation significance score of 0.1. We found 13 residues of EspB and 14 of EspK to contribute to the interface surface between both proteins (Fig. 3).

To further understand the EspB–EspK interaction, we performed SEC on EspK mutants in 1:1 mixtures with EspB. The EspB–EspK interaction could be abolished by either shortening an amino acid side chain of EspK through a K663A substitution or by changing the hydrophobicity of a side chain through a F659D substitution in EspK, indicating that both residues are essential for complex formation.

### Interaction with EspK prevents oligomerization of EspB

**Figure 4:**
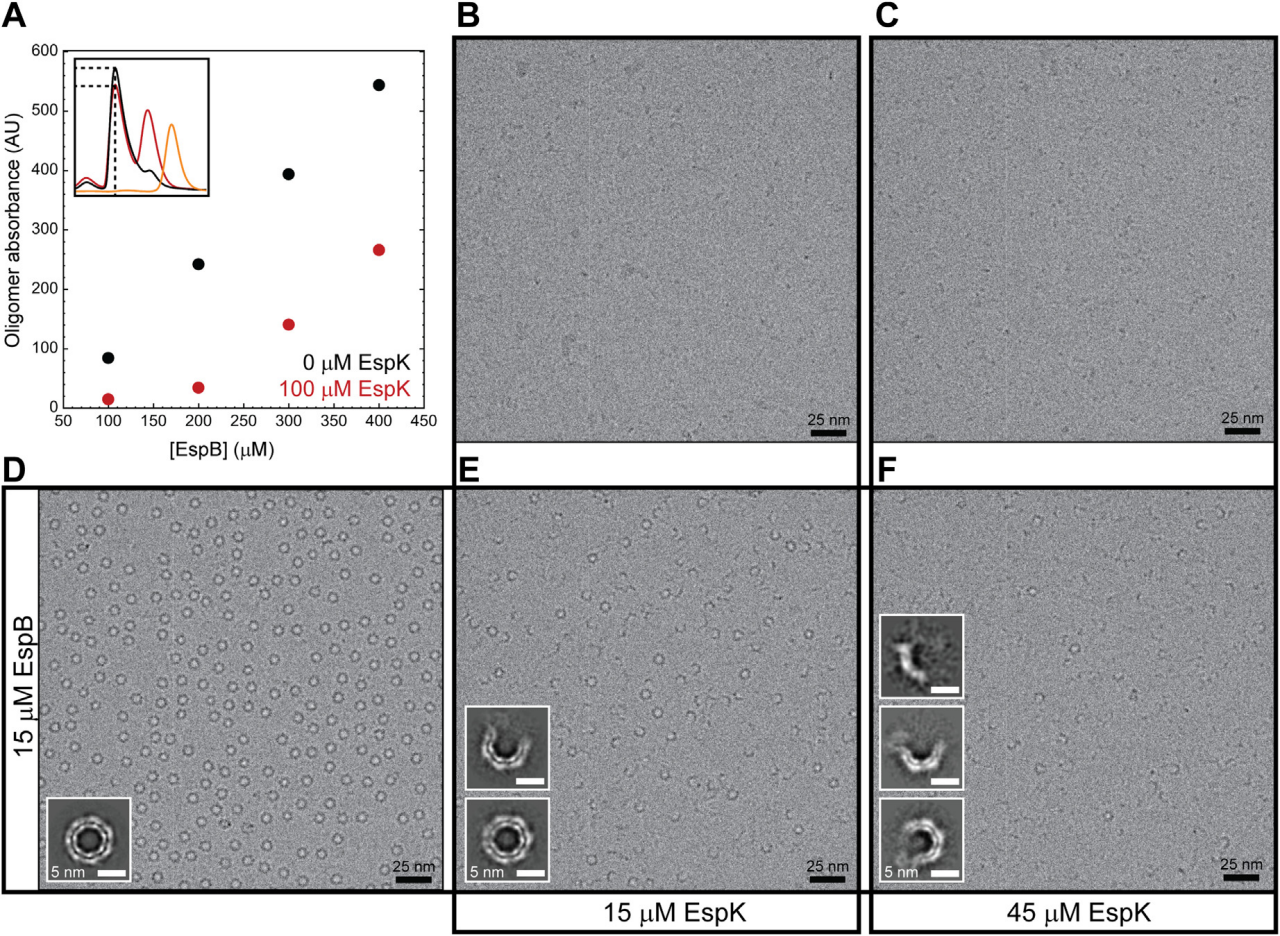
**Disruption of EspB oligomer in the presence of EspK.***A*, maximum absorbance of EspB oligomer at different concentrations, in the absence or presence of EspK. Inset corresponds to examples of profiles from where the maximum values were taken (EspB in *black*, EspB-EspK mixture in *red*, EspK in *yellow*, and dashed line EspB oligomer elution volume). Electron micrographs of EspK at (*B*) 15 μM and (*C*) 45 μM; (*D*) EspB at 15 μM; (*E*) mixture 1:1 molar ratio, and a (*F*) mixture 1:3 molar ratio. Insets correspond to the respective 2D classes.

EspK binds to the helical tip of EspB, as well as to a part of the PE-PPE loop. In earlier studies, we have demonstrated that the PE-PPE loop can adopt multiple confirmations and has to open up to allow for EspB to oligomerize (29). We showed previously that the position of the C-terminal end of this loop within the oligomeric state of EspB is incompatible with binding of EspK to EspB due to steric hindrance (Fig. 1, *C* and *D*). To demonstrate that EspK binding is not compatible with oligomerization of EspB, we evaluated the ability of EspB to form oligomers in the presence of EspK by SEC, cryo-EM, and SAXS. SEC experiments showed that the maximum absorbance of the peak corresponding to EspB oligomer was reduced when EspK was present compared to EspB alone, at all molar ratios tested (Fig. 4*A*). Cryo-EM sample preparation can be affected by many factors (sample thickness, air-water interface interaction time, blotting parameters, etc), which calls for caution when trying to use it to characterize complex formation. EspB oligomers adopt an extremely strong preferential orientation in cryo-EM (29), making the oligomer easy to visualize (Fig. 4*D*). EspK alone is too small to be seen by cryo-EM (Fig. 4, *B* and *C*). We looked at the effect of the addition of EspK on EspB oligomer formation by mixing EspB and EspK at 1:1 and 1:3 molar ratios and observing these samples by cryo-EM. Both molar ratios seem to disrupt the EspB oligomers in a concentration-dependent manner (Fig. 4, *E* and *F* and Experimental procedures). Addition of EspK to EspB did not solely result in a reduction of the number of preferred orientation ring-like particles in the micrographs but also in the appearance of new, broken oligomers, which were only seen in the mixtures (Fig. 4, *E* and *F*). We corroborated these findings through SAXS experiments coupled with online SEC. SEC-SAXS on EspB_2-348_ alone shows both heptamers as well as monomers (Fig. S3 black curve and Table S2). In the case of a mixture EspB_2-348_ and EspK_484-729_ (Fig. S3 red curve and Table S2), two peaks were observed. The first corresponds to a monomer complex, whereas the second peak on the right, corresponds to the excess of EspK, whereas no peak for the heptamer of EspB_2-348_ could be found. The combined findings of SEC, cryo-EM, and SAXS substantiates that presence of EspK competes with EspB oligomerization.

### EspB–EspK interaction is conserved in other mycobacterial species

The oligomerization of EspB is conserved for slow growing mycobacterial species (29). To investigate the importance of EspK to prevent EspB oligomerization, we assessed the interaction of this pair from two other mycobacterial species: *M. marinum*, another slow growing mycobacteria widely used as a model microorganism to study TB and probed to form an EspB oligomer, and *M*. *smegmatis*, a fast growing mycobacteria in which EspB does not oligomerize. SEC profiles showed that the interaction is conserved in both species (Fig. S4), suggesting that preventing oligomerization *an sich*, is not the primary function of EspK.

The C-terminal domain of EspK from *M. tuberculosis* has a high percentage identity with its ortholog from *M. marinum* (EspK: 80.4%). Thus, we tested if EspB and EspK proteins from *M. tuberculosis* could interact with their counterparts from *M. marinum*. As observed by a peak with an earlier elution volume in the SEC profiles, proteins from these evolutionary close species are able to interact (Fig. S5, *A* and *B*). However, none of the EspB and EspK proteins from *M. tuberculosis* and *M. marinum* interact with EspB and EspK from *M. smegmatis* (Fig. S5, *C*–*F*). This result may be due to the low sequence identity of EspK (53.1–53.3%) and EspB (28.5–30.7%) from *M. smegmatis* compared to those from *M. tuberculosis* and *M. marinum*. SEC experiments show an interaction between EspB and EspK from *M. smegmatis*.

**Figure 5:**
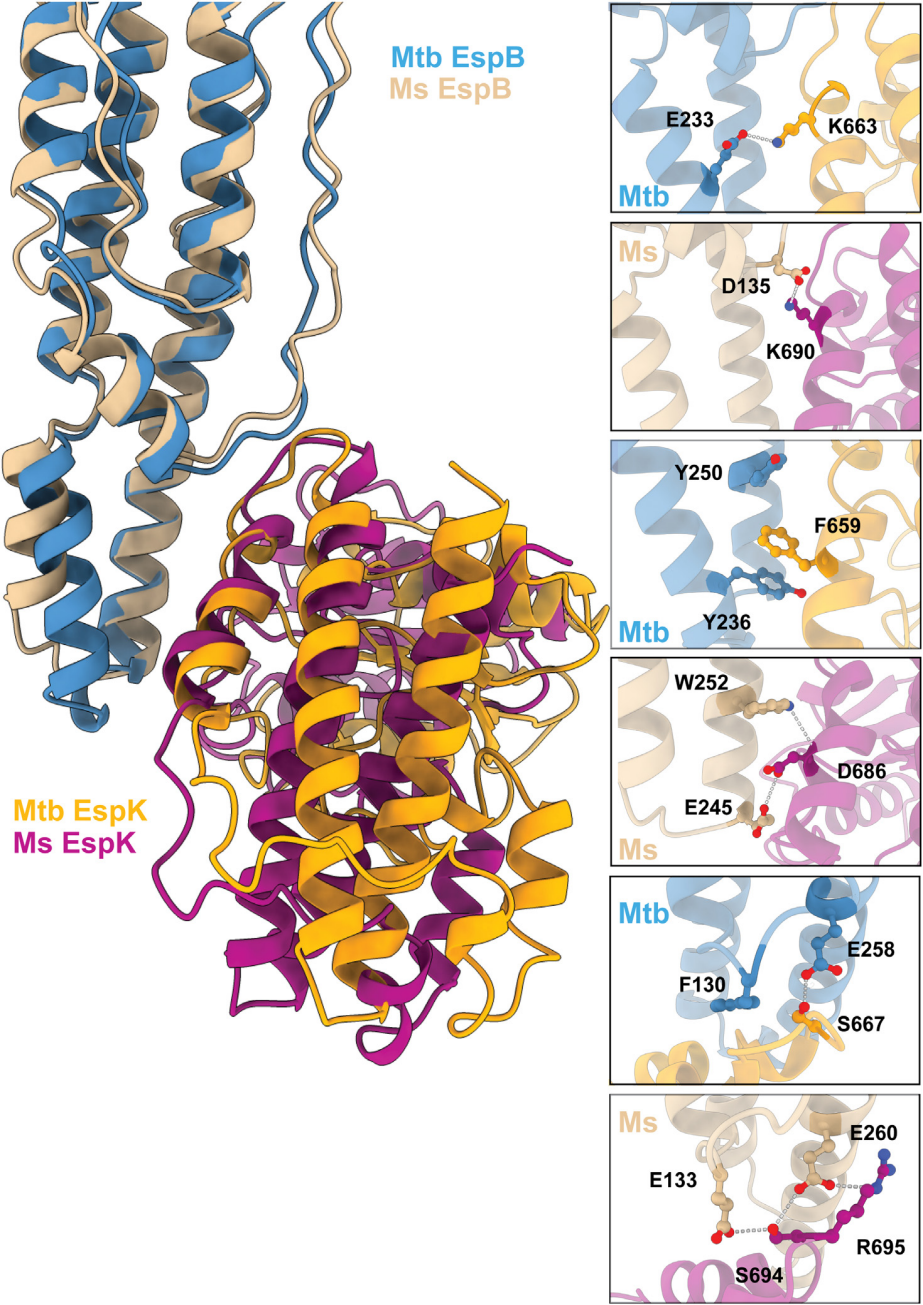
**Structural differences between EspB and EspK from *M. tuberculosis* and *M. smegmatis* in the complex context.** Crystal structure of EspB_2-300_ (*blue*) and EspK_484-729_ (*gold*) from *M. tuberculosis* aligned with a GalaxyWeb heterodimer prediction from EspB_1-311_ (*tan*) and EspK_510-755_ (*magenta*) from *M. smegmatis* (AlphaFold models A0A653FNI6 and A0QNK1). The inserts show some of the species-specific interactions. The distances for the polar interaction within our crystal structure between NZ of K663 and OE2 of E233 is 3.1 Å, whereas this distance is 2.6 Å for the interaction between OG of S667 and OE2 of E258. The predicted polar interactions (and their distances) within the GalaxyWeb heterodimer computational model for *M. smegmatis* are OD1 D135 - NH1 K690 (2.8 Å), NE1 W252 - O D686 (3.3 Å), OE2 E245 - OD1 D686 (3.3 Å), OE2 E133 - OG S694 (3.1 Å), and OE2 E260 - NE R695 (3.4 Å).

To obtain a molecular understanding of these results, we predicted the interaction between the homologous pair of *M. tuberculosis* EspB_2-300_ and EspK_484-729_ (hereafter named EspB_Mtb_ and EspK_Mtb_) within *M. smegmatis* (EspB_1__-311_ and EspK_510-755_; hereafter named EspB_Ms_ and EspK_Ms_) using a deep learning–based heteromer structure prediction method (46). The top solution found by the program seems to correspond to the global binding interface we observed for the *M. tuberculosis* pair (Fig. 5).

Analysis of the predicted *M. smegmatis* EspB-EspK by PISA (45) gave a larger interface area (831 *Å*^*2*^ compared to 652 Å^2^ for *M. tuberculosis*) but a much smaller solvation free energy gain upon formation of the interface (ΔG = -0.3 kcal/mol compared to -11.7 kcal/mol for *M. tuberculosis*), which could reflect inaccuracies in the predicted structures and interface, as we have experimentally shown that *M. smegmatis* EspB-EspK can form stable heterodimers. Closer inspection of the interacting residues reveals many fine differences between the *M. tuberculosis* and the *M. smegmatis* pairs (Fig. 5). A conserved lysine in EspK (K663 in EspK_Mtb_, K690 in EspK_Ms_) is predicted to jump from the observed interaction with E233 on the helical tip in EspK_Mtb_ to D135, which is located at the end of the PE-PPE loop in EspK_Ms_. A nearby conserved serine in EspK (S667 in EspK_Mtb_, S694 in EspK_Ms_) is observed to interact with E258 on the helical tip in EspB_Mtb_ and predicted to also interact with D135 in EspB_Ms_. E258 on the helical tip in EspB_Mtb_ is a conserved residue in EspB (E260 in EspB_Ms_); however, for the predicted structure of *M. smegmatis*, it is foreseen to interact with R695. More toward the end of the helical tip of EspB, the aromatic stacking observed for the *M. tuberculosis* pair (Y236 in EspB_Mtb_, F659 in EspK_Mtb_, and Y250 in EspB_Mtb_) is absent and replaced by hydrogen bonds in *M. smegmatis* (E245 in EspB_Ms_, D686 in EspK_Ms_, and W252 in EspB_Ms_) (Fig. 5).

It seems plausible that an EspK–EspB interaction could have happened in a shared ancestor, even earlier than EspB oligomerization, which suggests that blocking oligomerization is secondary to EspK’s main function. The lack of interaction between EspB and EspK proteins from the phylogenetic distant relative, *M. smegmatis*, with EspB or EspK from *M. marinum* and *M. tuberculosis*, suggests that these proteins have coevolved in order to maintain their interaction over time.

## Discussion

Despite arduous efforts to investigate the functioning of ESX-1, important information like the secretion process of protein substrates is still not fully understood and even completely missing for some substrates. One of these protein substrates is EspB. In earlier studies, we described the quaternary structure of EspB and the possible role that it could play as an element in the putative outer-membrane complex of ESX-1 (29). Knowing the importance of this protein in the proper functioning of the secretion system (31), we sought to gain further information on the secretion mechanism of EspB by studying its interaction with a putative chaperone, EspK.

EspB belongs to the PE/PPE family, a group of proteins that shares structural homology and comprise nearly 10% of the protein repertoire of pathogenic mycobacteria (47). This family of proteins is secreted by the T7SS with the assistance of the chaperone EspG; while all the systems secrete only one particular PE–PPE complex, ESX-5 secretes 95% of them (48). Despite their resemblance, the secretion of EspB does not depend on EspG_1_ (32). The interaction between EspB and the C-terminal domain of EspK, as well as the lack of EspB secretion in an *espK* mutant strain, led to the hypothesis that EspK was a possible chaperone of EspB (32). To better understand the relationship between these proteins, we solved the crystal structure of the C-terminal domain of EspK bound to EspB. The complex structure revealed that EspK binds to the helical tip of EspB, a feature shared with its PE–PPE homologs and their chaperone EspG (37, 42, 43). The interaction at this position leaves the WxG and YxxxD motifs in EspB accessible for the interaction with the ESX-1 secretion system (29).

**Figure 6:**
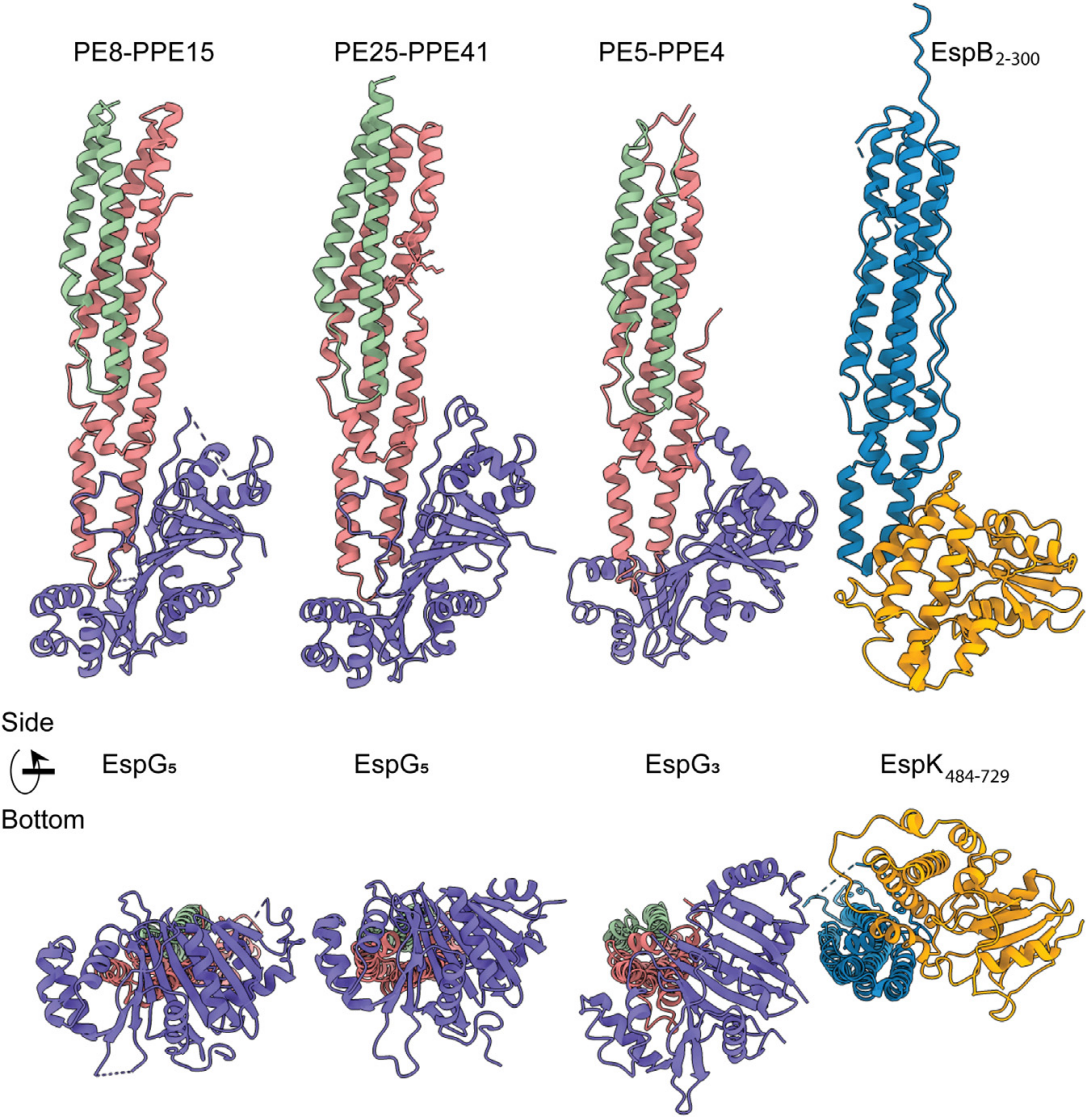
**EspG and EspK bind to the helical tip of their respective binding partner.** Structure comparison of the PE-PPE-EspG ternary complexes available in the literature (PDB ID: 5XFS, 4KXR, 6UUJ) and the EspB–EspK complex. PE’s are colored in *green*, PPE’s in *pink*, EspG’s in *purple*, EspB in *blue*, and EspK in *gold*. PDB, Protein Data Bank.

To date, the structures of EspG_1_, EspG_3_, and EspG_5_ have been solved, and although, they present the lowest sequence identity of all the paralogs in the ESX-systems (13–23%), their tertiary structure is conserved (49). However, they show enough structural differences to present secretion specificity (Fig. 6, (37)). Two key regions have variances that alter the shape of the PPE-binding surface, which may translate into binding specificity: the β2-3 loop changes in length and structure and the α2 in EspG_3_ is longer, which would clash with the PPE protein in the EspG_5_ orientation (37). EspK does not share any structural homology with EspG; however, both proteins bind the helical tip of different members of the PE-PPE family (Fig. 6).

Chaperones involved in secretion systems play multiple functions to keep proteins in a secretion-competent state: stabilization of insoluble proteins, preventing premature folding or self-oligomerization, directing substrates to the secretion machinery, and regulating the hierarchy in the secretion process (37, 50, 51, 52). It has been shown that the interaction with EspG keeps PE–PPEs soluble (37, 53). EspB is a highly soluble protein and does, unlike most PE-PPE proteins, not require a chaperone to keep it soluble. Instead, EspK interferes with the oligomerization of EspB, which supports the idea that this interaction occurs in the cytosol of the bacterium. Despite what could seem obvious, the location of the interaction has never been tested. It was suggested to occur in the cytosol because McLaughlin *et al.* (32) could not find EspK secreted into the medium. However, since then, multiple studies have shown the presence of EspK in the capsule or culture filtrate (38, 54, 55). Recently (39), it was shown that EspK is exported by *M. tuberculosis* to be localized in its cell wall. As EspB oligomerizes upon secretion, it is expected that any control on this event would happen before its secretion. We hypothesize that EspK binds to EspB to prevent a premature oligomerization that could stop its secretion (39). We have shown that one of the factors that favors EspB oligomerization is cleavage of its C-terminal region, which occurs during secretion within the periplasm, as well as the presence of an acidic environment (29). Nevertheless, oligomerization of EspB full length could also happen in the not so basic environment of the mycobacterial cytoplasm. So by blocking EspB oligomerization, EspK can ensure that EspB gets through the secretion machinery, making the process more efficient.

**Figure 7:**
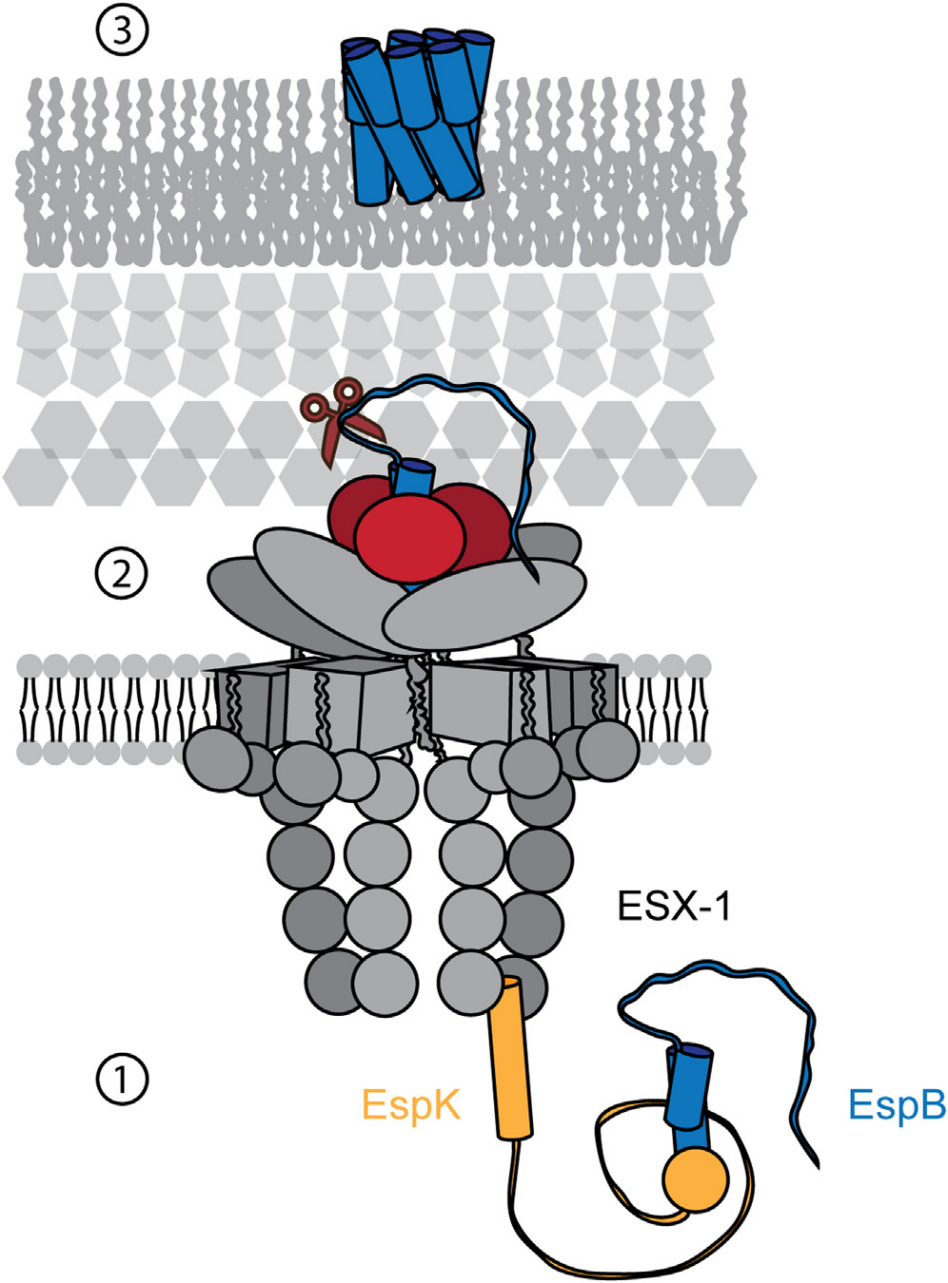
**Scheme of suggested secretion pathwayof EspB.** (1) EspB interacts with the C-terminal domain of EspK, which pulls it toward ESX-1 to be recognized by the machinery (2). EspB is secreted over the inner membrane by ESX-1 and cleaved by MycP_1_ at its C-terminal region (3). EspB meets the conditions to oligomerize and be functionally active.

It has been suggested that EspK directs EspB to the ESX-1 machinery by connecting EspB to EccCb_1_ (32). A model where the N-terminus of EspK binds to EccCb_1_ and the C-terminus binds to EspB was described in *M. marinum* and *M. tuberculosis*, the latter having been confirmed here. However, they only included an interaction with EccCb_1_ through the N-terminus of EspK in their model, even though their data also showed an interaction with its C-terminus (see Fig. S3*B* of (32)). In the same study, they could not detect an interaction between EspB and EccCb_1_, while Solomonson *et al.* (2015) saw a weak interaction in *M. smegmatis*. The secretion of substrates via the T7SS is defined by a bipartite signal (YxxxD/E and WxG motifs), and although the integrity of those motifs have been proved essential (26), no interaction of this motif has been described so far with this machinery or any other element (54, 56, 57). On the other hand, EccCb_1_ recognizes hydrophobic residues seven positions downstream of the YxxxD/E motif in the EsxB substrate (56, 57). Sequence alignment on multiple ESX-secreted proteins, including EspB, suggests that this is a conserved characteristic and the name “export arm” was assigned to this region (34). It is noteworthy that in EspB from *M. smegmatis*, the export arm is an extension of the α2 (34), while in *M. marinum* and *M. tuberculosis*, it is unstructured (29, 35), which might explain the difference in the aforementioned information. Taken all together, we suggest the following putative model of the secretion mechanism of EspB (Fig. 7): EspK recruits EspB through its C-terminal domain to maintain a monomeric form, while its N-terminal domain contacts the EccCb_1_ export machinery. The long and flexible linker that connects such domains (40) works as a rope pulling the C-terminal domain–EspB complex toward the secretion machinery. The interaction between EspB and EccCb_1_ allows the recognition of EspB’s export arm. Next, secretion of EspB through the inner membrane occurs, after which it could form an oligomeric structure that allows the transit of proteins toward and through the outer membrane (Fig. 7).

In conclusion, we have characterized the *M. tuberculosis* structure of EspB in complex with its chaperone EspK. The C-terminal globular domain of EspK interacts with the helical tip as well as the end of the PE-PPE loop of EspB. EspK can keep EspB in a monomeric state by avoiding premature oligomerization. The structure of EspB–EspK indicates a secretion mechanism different from the ones assisted by EspG, illustrating the complexity of the structure and mechanism of ESX secretion. The structure of EspK represents an opportunity to design therapeutic compounds that could interact with EspB, disrupt EspB oligomers, and possibly diminish virulence of mycobacteria.

## Experimental procedures

### Protein expression and purification

Different constructs used in this study are listed in Table S1. EspK constructs from *M. smegmatis* and *M. marinum* were synthesized and codon optimized for expression in *Escherichia coli* (Genscript). They were designed based on the *M. tuberculosis* construct. EspB constructs from all organisms, as well as EspK from *M. tuberculosis*, were expressed in Rosetta 2(DE3) *E. coli* and purified by conventional chromatographic techniques as described previously (29). For the codon-optimized constructs, proteins were expressed in C41(DE3) *E. coli* cells following the aforementioned procedure.

### Analytical SEC

Samples were dialyzed overnight in 20 mM Tris–HCl (pH 8.0), 150 mM NaCl, loaded onto a size-exclusion Superdex 200 Increase 3.2/300 column (GE Healthcare Life Science) and eluted with the same buffer at a flow rate of 50 μl/min.

### SAXS experiments

SAXS experiments (Table S2) on the EspB constructs were originally collected in the bioSAXS beamline P12-EMBL at DESY Light Source (58). Sample injections of 50 μl were run on a Superdex 200 Increase 5/150 GL size-exclusion column attached to a FPLC–Malvern TDA system at a flow rate of 0.1 ml/min. The elution output was directed through a quartz capillary cell (50 μm thick wall and a 1.7 mm path length) held in vacuum. Data acquisition consisted of 900 frames (with 1 s exposure time) using a PILATUS 2M detector at the distance of 3.0 m from the sample. Images were corrected for variations in beam current, normalized for time exposure, and processed into one-dimensional scattering curves using integrated software at the beamline (59). Background subtraction was done with the program CHROMIXS (60).

SAXS data on EspB and EspK were collected in the bioSAXS beamline B21 at Diamond Light Source (61). Experiments were performed coupled to a Shodex KW-403 SEC column equilibrated with 20 mM Tris–HCl (pH 8.0), 300 mM NaCl. Injections of 50 μl were made. EspB–EspK mixtures were used in 1:5 molar ratio. The eluted protein was directed through a 1.6 mm diameter quartz capillary cell held in vacuum. Data acquisition consisted of 580 frames (with 3 s exposure time) using a PILATUS 2M detector at a calibrated distance of 4.014 m from the sample. Images were corrected for variations in beam current, normalized for time exposure, and processed into one-dimensional scattering curves using GDA and the DAWN software (Diamond Light Source). SAXS parameters are listed in Table S2. Missing flexible regions were added to the crystal models using the CORAL program to improve fitting (62).

### Structure determination by macromolecular crystallography

#### Crystallization conditions

EspB (residues 2–348) or EspB (residues 2–300) were incubated together with EspK (residues 484–729) from *M. tuberculosis* for 30 min at 4 °C. The EspB–EspK protein complex was then isolated by gel filtration using a Superdex 200 Increase 10/300 GL column and concentrated to 8 mg/ml in 50 mM Tris–HCl (pH 8.0) and 300 mM NaCl. Protein concentration of the complex mixture was determined by 280 nm absorbance using an extinction coefficient of 63,495 M^-1^ cm^-1^ theoretically calculated with ExPASy.

Initial crystallization conditions were identified at the High Throughput Crystallization Facility, EMBL Grenoble, France. The best condition for the EspB (residues 2–348) and EspK (residues 484–729) complex was obtained from the JCSG-Plus (Molecular Dimensions) condition F10 (1.1 M sodium malonate, 0.1 M Hepes, pH 7.0, and 0.5% (v/v) jeffamine ED-2003), with a high solvent content (>70 %) and anisotropic diffraction to ∼4.6 Å. Manual optimization and screening of various additives, with PEG3350 in particular identified, improved the crystal quality. Later, because the C-terminal region of EspB (residues 2–348) was observed to be disordered in initial maps, a shorter EspB (residues 2–300) construct was designed to further improve the diffraction quality. This EspB (residues 2–300) and EspK (residues 484–729) complex crystallized in similar conditions and produced the best diffracting crystals with reduced anisotropy. The best diffracting crystal conditions were produced by mixing 1 μl of the concentrated EspB–EspK complex (8 mg/ml) with 1 μl of a reservoir solution in a 24-well plate (0.2–1.2 M sodium malonate, 0.1 M Hepes (pH 7.8), and 8% to 14% PEG3350). These crystals were cryoprotected by supplementing the mother liquor with 25% glycerol, mounted onto a nylon loop, and flash frozen in liquid nitrogen until data collection.

For experimental phasing (Hg-SAD: data were collected prior to the release of AlphaFold Protein Structure Database (63)), crystals were grown in a slightly different condition (0.2–1.2 M sodium malonate, 0.1 M Hepes (pH 7.8)) and soaked in a solution corresponding to the mother liquor, supplemented with 25% glycerol and 5 mM p-Chloromercuribenzoic acid (Hampton HR2-446-08), previously dissolved in dimethyl sulfoxide as a 200 mM stock solution. Crystals were mounted onto a nylon loop and flash frozen in liquid nitrogen until data collection.

### Data collection, analysis, and structure refinement

The highest resolution native and S-SAD data were collected on ID30B at the European Synchrotron Radiation Facility (64). Hg–derivative data were collected on EMBL beamline P13 at the PETRA III storage ring (DESY) (65). All data were integrated using the XDS suite (66). The diffraction data were highly anisotropic, with diffraction limits of ∼2.3 Å, 2.9 Å, and 2.8 Å along the best direction for native, S-SAD, and Hg derivative, respectively, but only ∼3.1 Å, 3.6 Å, and 4 Å in the weakly diffracting direction. Therefore, all data were processed using STARANISO, as implemented in autoPROC (67), which is comprised of three main steps. Firstly, an analysis of the nonelliptical anisotropy decay of the mean intensities (I) was performed. This anisotropy was then taken into account in the second step by a Bayesian estimation of structure factor amplitudes that was applied to small or negative intensities. In the last step, the decay of local averaged mean I/σ(I) in different directions was analyzed to provide a basis for the anisotropic resolution limit at a cutoff level of 1.2 I/σ(I).

The EspB_2-300_–EspK_484-729_ complex structure was solved by molecular replacement with Phaser (68), using the EspB structure (PDB ID: 4XXX) as a search model (35). The experimental phases were further improved, and a preliminary model for EspK was generated using the Phenix AutoBuild module (69). The model was completed by cycles of manual building in Coot (70) and refinement rounds in BUSTER, Phenix (69), Refmac5 (71), and PDB-REDO (72). MolProbity (73) was used for model validation. The final model has been deposited in the Protein Data Bank as PDB entry 8AKO.

### Cryo-EM

Proteins were dialyzed against fresh 20 mM Tris (pH 8.0) and 150 mM NaCl prior to the experiment. EspB_2–348_ (final concentration of 15 μM) was mixed with EspK_484-729_ in a molar ratio of 1:1 and 1:3. Mixtures and individual proteins, as controls, were vitrified by applying 2.5 μl of sample on a glow-discharged UltrAuFoil Au300 R1.2/1.3 grids (Quantifoil), excess liquid removed by blotting for 3 s (blot force 5) using filter paper, and plunge freezing in liquid ethane using a FEI Vitrobot Mark IV at 100% humidity at 4 °C. Cryo-EM data were collected using EPU (Thermo Fisher Scientific) on an Arctica Tecnai (Thermo Fischer Scientific) at 200 kV with a Falcon III detector in integration mode. All EPU data were collected with a requested defocus of 1 μm underfocus, a nominal magnification of 110k×, 3.1 s exposure time, and a total fluence of 50 e^-^/Å^2^. Data were processed using the RELION pipeline (74); however, as only qualitative information was looked for, until up to 2D classification. Movie stacks were corrected for drift (5 × 5 patches) and dose-weighted using MotionCor2 (75). The local contrast transfer function parameters were determined for the drift-corrected micrographs using Gctf (76). Particle picking was done iteratively, starting with manual picked samples from the EspB alone datasets. These were combined into 2D classes, of which the best two were chosen for two subsequent rounds of template-based Autopicking, particle extraction, 2D classification, and two best 2D classes selection. These resulted in 2D classes of both complete and incomplete ring-shape oligomers of ∼10 nm diameter. A total of 152 micrographs were collected for the EspB-alone sample, 195 micrographs for the EspB-EspK 1:1 sample, and 137 micrographs for the EspB-EspK 1:3 sample. These resulted in 31,405, 5712, and 5652 picked particles for the EspB-alone, EspB-EspK 1:1, and EspB-EspK 1:3 sample, respectively, corresponding to an average of, respectively, 207, 29, and 41 particles per micrograph.

### Bioinformatics and modeling

The Dali server (77) was used to identify proteins with similar folds of EspK_484-729_. The PDBePISA server (45) was used to explore the binding interface between EspB and EspK. EspB and EspK predicted structures for *M. smegmatis* (A0A653FNI6 and A0QNK1) were obtained from the AlphaFold Structure Prediction Server (63, 78) and truncated to 1 to 311 for EspB_Ms_ and 510 to 755 for EspK_Ms_. These structures were provided to the GalaxyWEB webserver for protein structure heteromer prediction (GalaxyHeteromer) (46). The top 10 heterodimer solutions were downloaded and manually inspected.

## Data availability

The crystallographic model has been deposited in the Protein Data Bank as entry 8AKO. The SAXS data have been deposited in the Small Angle Scattering Biological Data Bank (SASBDB (79)) under the accession numbers SASDMD7 (EspB_2-406_), SASDME7 (EspB_2-348_ monomer), SASDQF4 (EspB_2-348_ heptamer), SASDMF7 (EspB_7-278_), SASDMG7 (EspK_484-729_ + EspB_2-406_), SASDMH7 (EspK_484-729_ + EspB_2-348_), and SASDMJ7 (EspK_484-729_ + EspB_7-278_).

## Supporting information

This article contains supporting information.

## Conflict of interest

The authors declare that they have no conflicts of interest with the contents of the article.
