# Supplementary material for: The crystal structure of the EspB-EspK virulence factor-chaperone complex suggests an additional type VII secretion mechanism in Mycobacterium tuberculosis
Source: J Biol Chem. 2022 Dec 1;299(1):102761. doi: 10.1016/j.jbc.2022.102761 (PMC9811218; doi:10.1016/j.jbc.2022.102761)
Supplement: Supporting information [file mmc1.docx]

# Supporting information


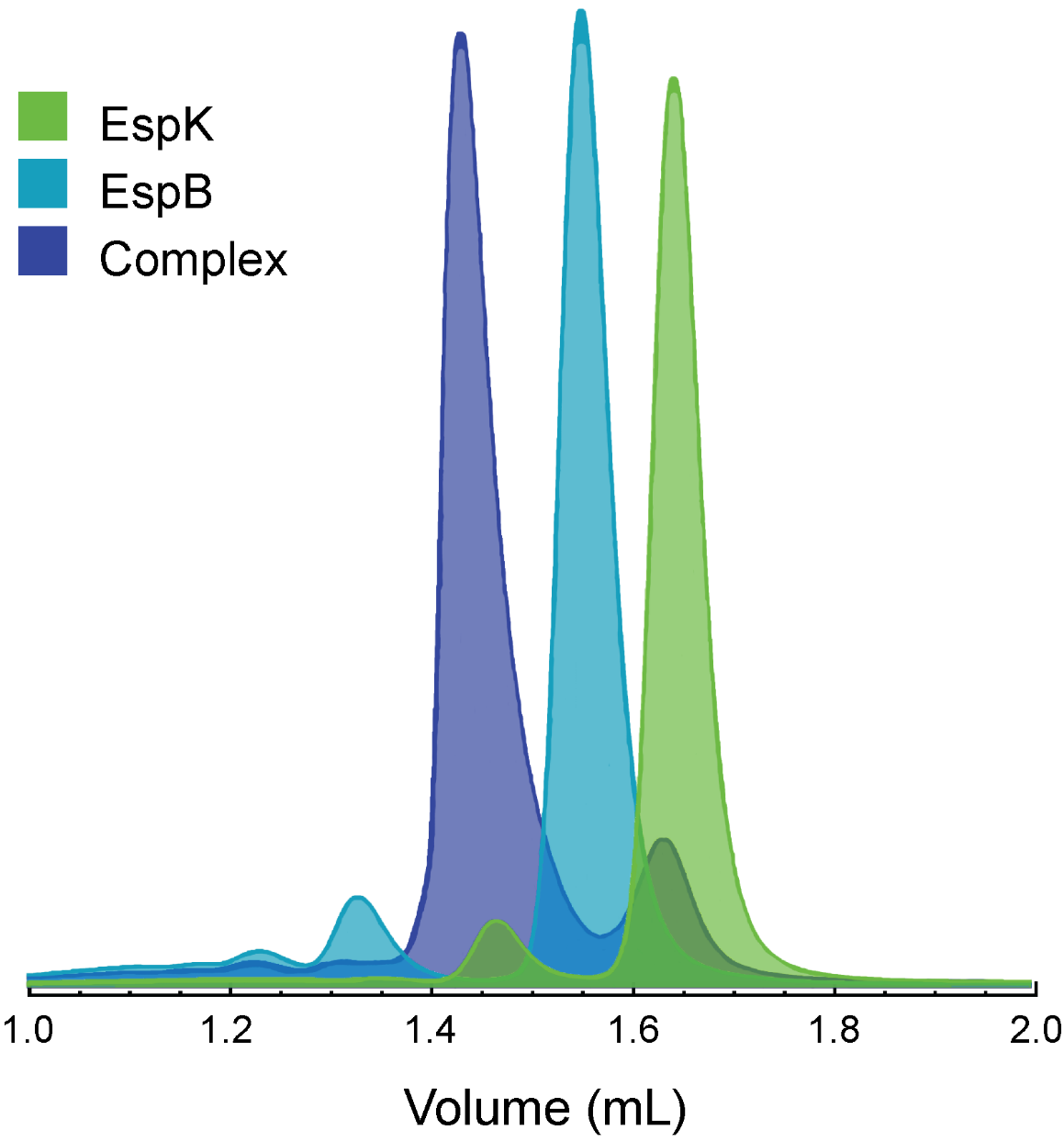


**Supplementary Figure 1. EspB and EspK form a complex.** Size-exclusion chromatography profiles of *M. tuberculosis* EspK_484-729_, EspB_7-278_, and a mixture of 1:1 molar ratio (labelled Complex). Experiments were performed at 100 µM in 20 mM Tris-HCl (pH 8.0), 150 mM NaCl. Void volume correspond to 0.8 ml elution volume.


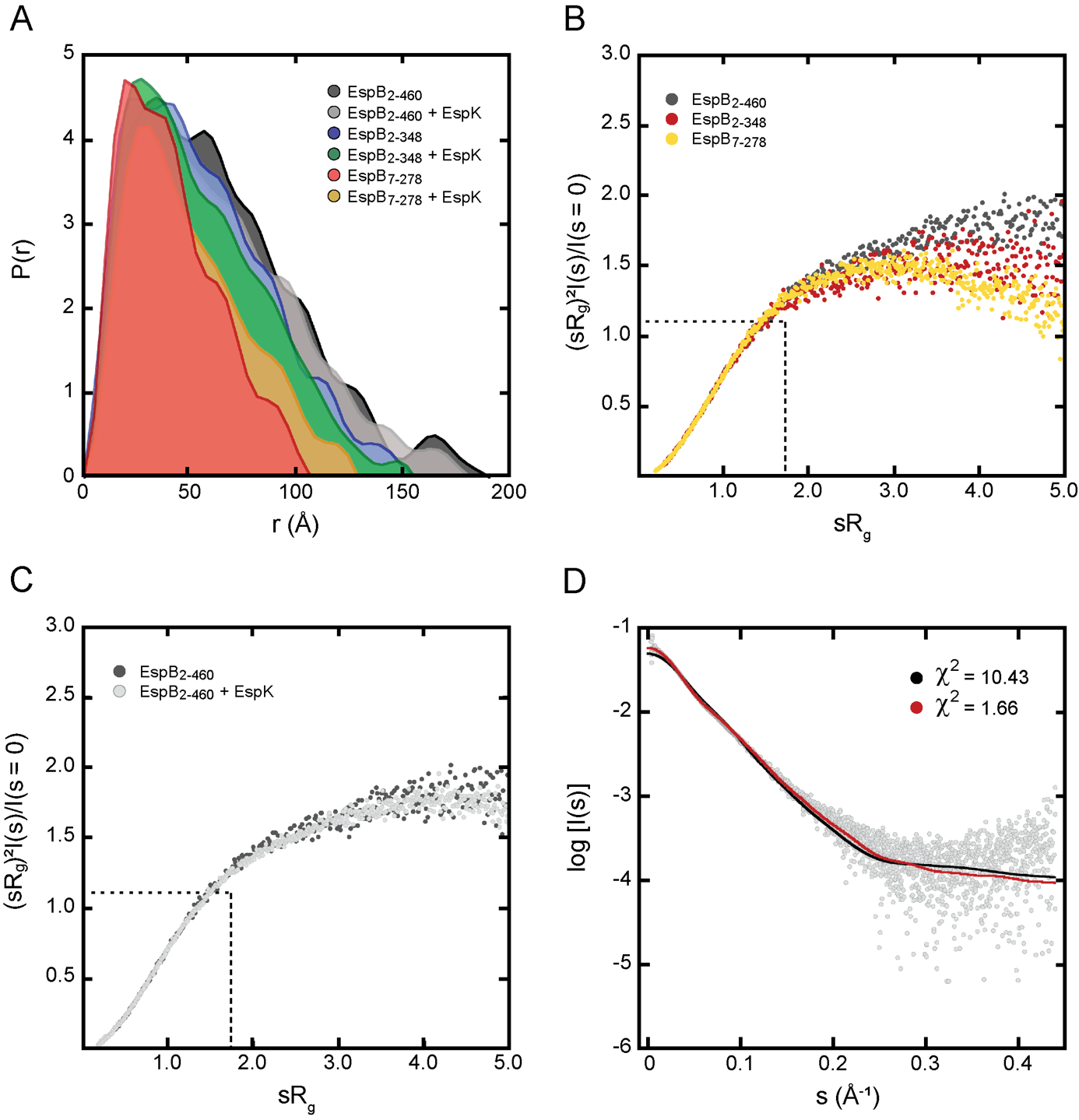


**Supplementary Figure 2. Low-resolution SAXS data suggests N-terminal region of EspB solely binds EspK.** (A) Pair distribution function plot and dimensionless Kratky plot of EspB constructs in the (B) absence or (C) presence of EspK. In B and C, the intersection of the dotted black trace corresponds to the value for the reference protein bovine serum albumin (BSA). (D) Fit of the calculated SAXS scattering curves of the crystal complex structure (EspB_2-300_ – EspK_484-729_) compared to the experimental scattering signal (black line). As experimental SAXS data was done on EspB_2-348_- EspK_484-729_, 48 residues were added to the EspB crystal structure by the program CORAL (Petoukhov et al. 2012) to make them comparable (red line).


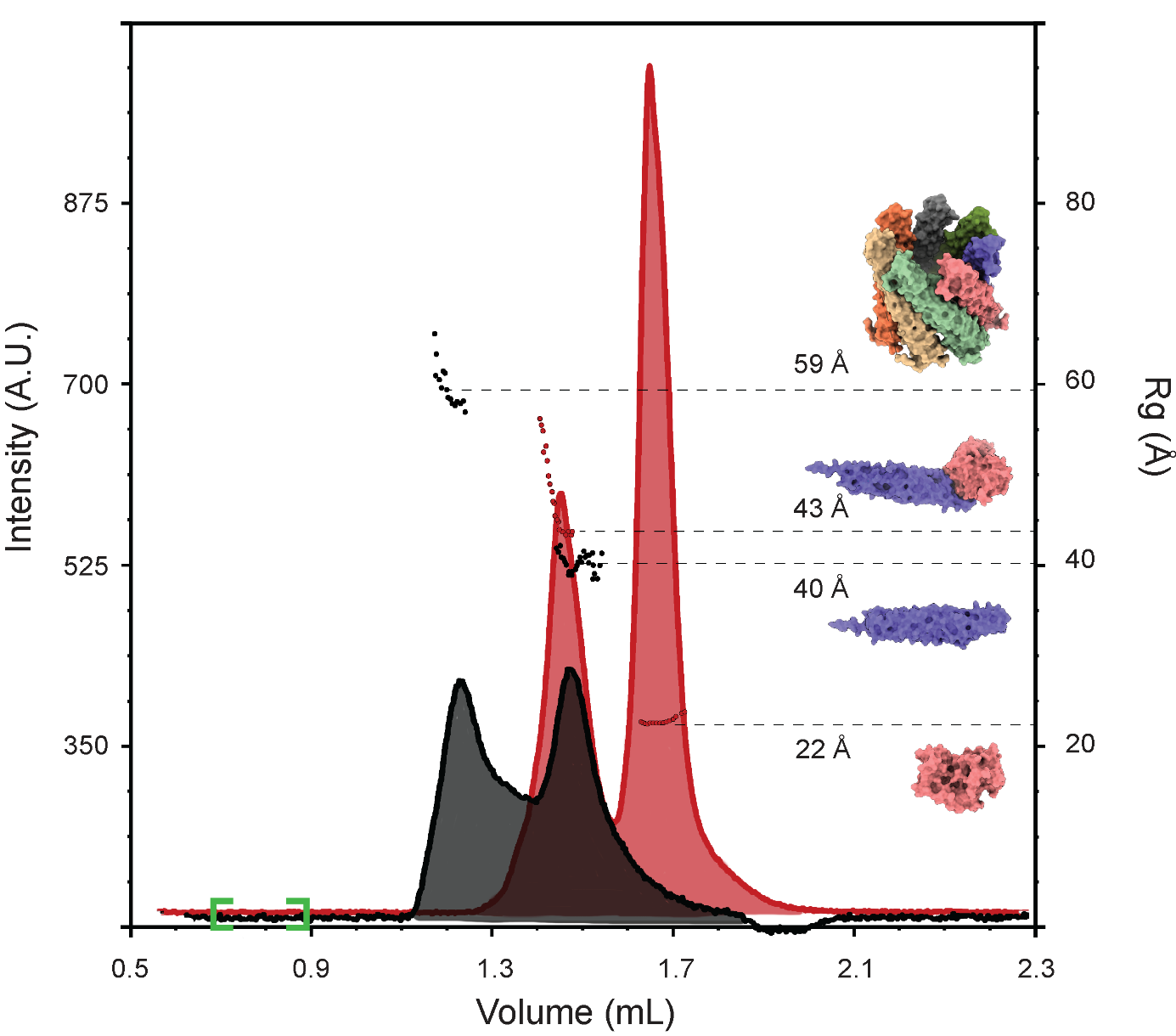


**Supplementary Figure 3. SEC-SAXS experiments show no EspB oligomer in the presence EspK.** Integrated scattering intensity and radius of gyration of EspB_2-348_ (black) or the mixture of EspB_2-348_ and EspK_484-729_ (red) (molar ratio 1:3). Radius of gyration calculated from Guinier approximation and the molecule that correspond to it (dashed line). Green brackets correspond to the area used to remove the background signal from the buffer.


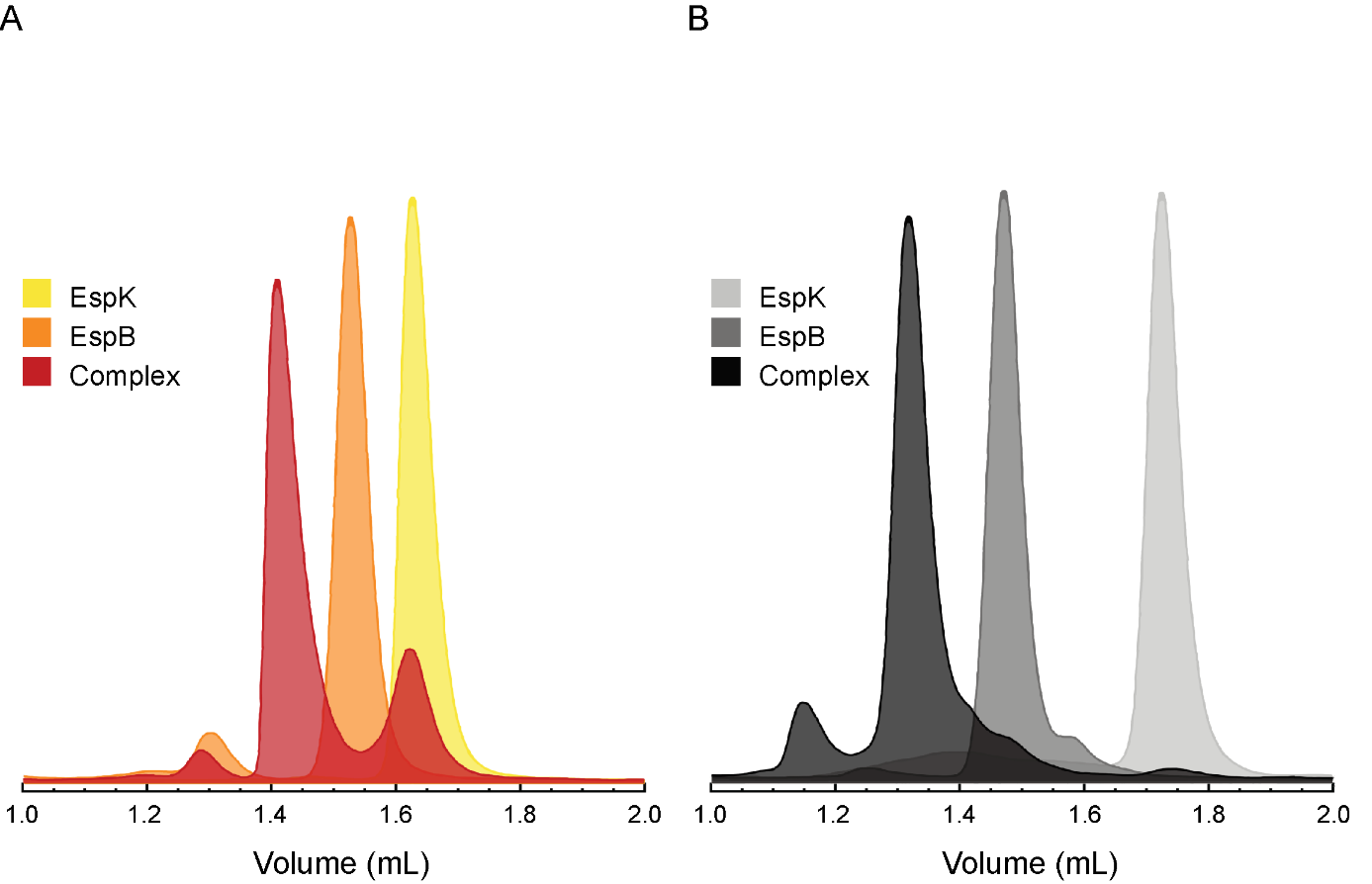


**Supplementary Figure 4. EspB and EspK from other species also form a complex.** (A) Size-exclusion chromatography profiles of (A) *M. marinum* EspK_532-776_, EspB_2-286_, and a mixture of 1:1 molar ratio (labelled Complex) and of (B) *M. smegmatis* EspK_515-755_, EspB_2-407_, and a mixture of 1:1 molar ratio (labelled Complex). Experiments were performed at 100 µM in 20 mM Tris-HCl (pH 8.0) and 150 mM NaCl. Void volume correspond to 0.8 ml elution volume.


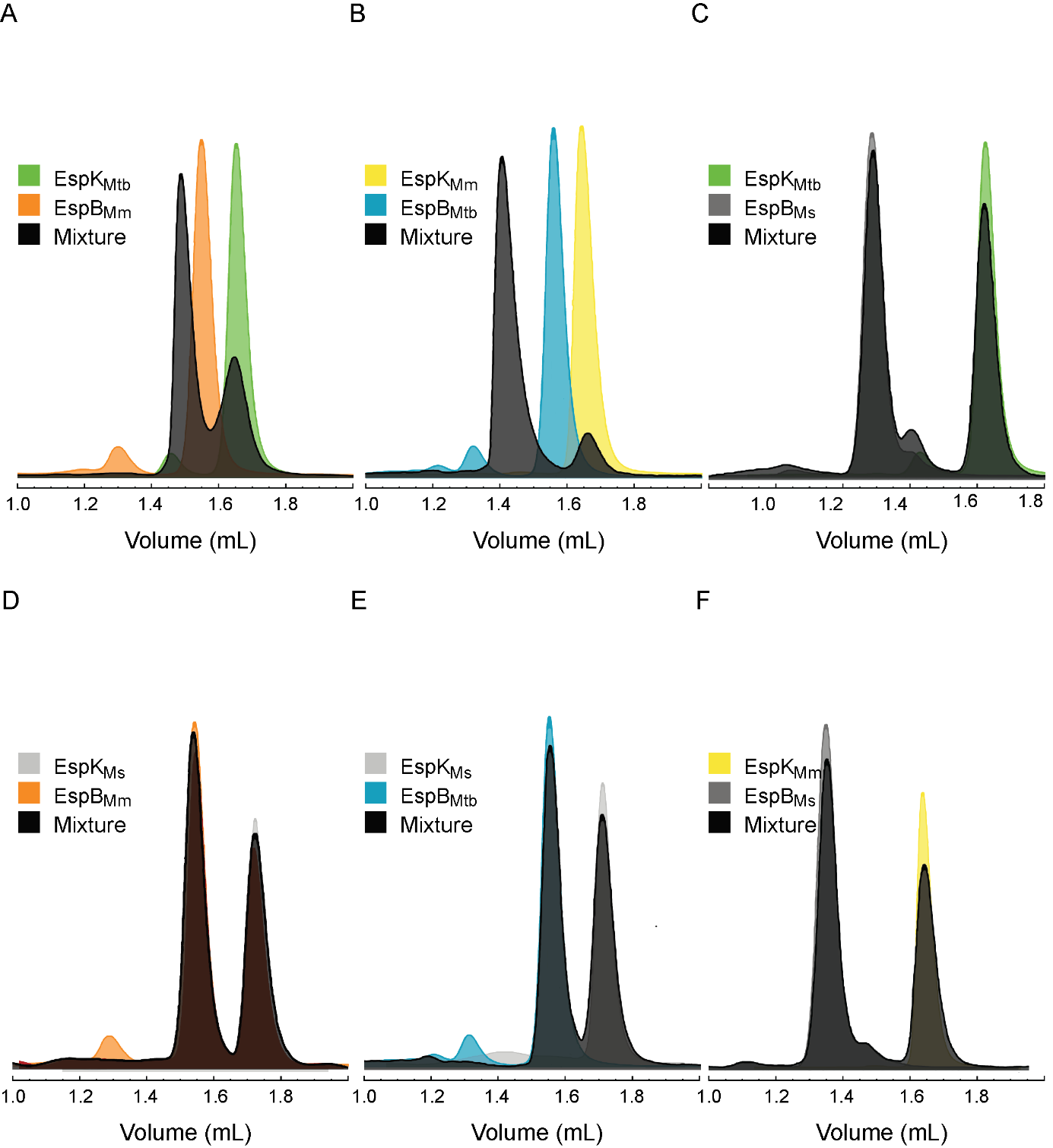


**Supplementary Figure 5. No EspB and EspK inter-species interaction is formed with *M. smegmatis*.** Size-exclusion chromatography profiles of EspK, EspB, and a mixture of 1:1 molar ratio. (A) *M. tuberculosis* EspK_484-729_ and *M. marinum* EspB_2-286_. (B) *M. marinum* EspK_532-776_ and *M. tuberculosis* EspB_7-278_. (C) *M. tuberculosis* EspK_484-729_ and *M. smegmatis* EspB_2-407_. (D) *M. smegmatis* EspK_515-755_ and *M. marinum* EspB_2-286_. (E) *M. smegmatis* EspK_515-755_ and *M. tuberculosis* EspB_7-278_. (F) *M. marinum* EspK_532-776_ and *M. smegmatis* EspB_2-407_. Experiments were performed at 100 µM in 20 mM Tris-HCl (pH 8.0) and 150 mM NaCl. Void volume correspond to 0.8 ml elution volume.

**Table S1.** Constructs from this study and the experiments in which they were used.

| Specie | Construct | Experiment |
| --- | --- | --- |
| *M. tuberculosis* | EspB 2-460 | SAXS and MX |
| *M. tuberculosis* | EspB 2-348 | SAXS and cryo-EM |
| *M. tuberculosis* | EspB 2-300 | MX |
| *M. tuberculosis* | EspB 7-278 | SEC and SAXS |
| *M. tuberculosis* | EspK 484-729 | SEC, MX, SAXS and cryo-EM |
| *M. marinum* | EspB 2-286 | SEC |
| *M. marinum* | EspK 532-776 | SEC |
| *M. smegmatis* | EspB 2-407 | SEC |
| *M. smegmatis* | EspK 515-755 | SEC |

Table S2. SAXS data collection and experimental parameters for *M. tuberculosis* EspB constructs alone and in complex with the C-terminal domain of EspK.

| Data collection parameters | P12, DESY (Hamburg, Germany) | | | | | B21, Diamond Light Source (Harwell, UK) | | |
| --- | --- | --- | --- | --- | --- | --- | --- | --- |
| **Detector (distance to sample)** | Pilatus 6M (3000 mm)  0.2 × 0.3 mm  10.0 kV  0.0038 – 0.42  1  900  293  SEC online | | | | | Pilatus 2M (4014 mm) | | |
| **Beam size** |  |  |  |  |  | 0.2 × 0.2 mm | | |
| **Energy** |  |  |  |  |  | 12.4 kV | | |
| ***q* range (A^−1^)** |  |  |  |  |  | 0.0038 – 0.42 | | |
| **Exposure time (s)** |  |  |  |  |  | 3 | | |
| **Number of frames** |  |  |  |  |  | 620 | | |
| **Temperature (K)** |  |  |  |  |  | 293 | | |
| **Mode** |  |  |  |  |  | SEC online | | |
| Structural parameters | | **EspB_2-460_** | **EspB_2-348_**  **_heptamer monomer_** | | **EspB_7-278_** | **EspK_484-729_** –  **EspB_2-460_** | **EspK_484-729_** –  **EspB_2-348_** | **EspK_484-729_** –  **EspB_7-278_** |
| **Injection concentration (mg/ml)** | | 6.1 | 5.6 | | 6.7 | 4.3/1.5 | 8.6/2.4 | 8.6/1.9 |
| ***q* Interval for Fourier inversion (Å^−1^)** | | 0.009–0.159  54.70 ± 0.26  50.10 ± 0.04  0.45–1.28  191  119  52–59 (92%)  48 | 0.006–0.135 | 0.009–0.200 | 0.011–0.251 | 0.008–0.216 | 0.011–0.184 | 0.016–0.195 |
| ***R_g_* [from P(r)] (Å)** | |  | 58.77 ± 0.33 | 42.60 ± 0.12 | 34.11 ± 0.21 | 54.90 ± 0.42 | 45.24 ± 0.42 | 42.94 ± 0.12 |
| ***R_g_* [from Guinier approximation] (Å)** | |  | 59.18 ± 0.52 | 40.00 ± 0.04 | 32.60 ± 0.17 | 49.60 ±0.19 | 43.24 ± 0.12 | 40.77 ± 0.13 |
| ***sR_g_* limits [from Guinier approximation]** | |  | 0.37–1.28 | 0.40–1.30 | 0.27–1.25 | 0.42–1.30 | 0.40–1.30 | 0.67–1.30 |
| **Dmax (Å)** | |  | 187 | 157 | 108 | 183 | 157 | 130 |
| **Porod volume estimate (nm^3^)** | |  | 625 | 75 | 56 | 128 | 102 | 113 |
| **Molecular Mass (kDa)** | |  |  |  |  |  |  |  |
| **Credibility interval (probability)** | |  | 264-318 (100%) | 41–46 (91%) | 34–39 (92%) | 71–78 (94%) | 61–69 (94%) | 53–59 (92%) |
| **From sequence** | |  | 261 | 37 | 30 | 75 | 64 | 57 |
| **SASBDB code** | | SASDMD7 SASDQF4 SASDME7 SASDMF7 | | | | SASDMG7 SASDMH7 SASDMJ7 | | |
| Software employed | |  | | | | | | |
| **Primary data reduction** | | DAWN pipeline (Diamond Light Source, UK)  ScÅtter v3.1q, ATSAS  CORAL  CRYSOL | | | | | | |
| **Data processing** | |  |  |  |  |  |  |  |
| **Modelling** | |  |  |  |  |  |  |  |
| **Computation of model intensities** | |  |  |  |  |  |  |  |
